# A chain multiple mediation model reveals the association between abuse and depression in Chinese adolescents

**DOI:** 10.3389/fpubh.2022.1023749

**Published:** 2022-11-17

**Authors:** Xiaoqing Zhan, Kuiliang Li, Yingcan Zheng, Guoyu Yang, Xi Luo

**Affiliations:** ^1^Department of Medical English, School of Basic Medical Sciences, Army Medical University, Chongqing, China; ^2^School of Psychology, Army Medical University, Chongqing, China

**Keywords:** depression, academic stress, social anxiety, generalized anxiety, abuse

## Abstract

**Background:**

Depression is a common mental disorder in Chinese adolescents. Identifying its risk factors will facilitate early prevention. As abuse is reported to be a great risk factor of depression, it is necessary to identify factors mediating the relation between abuse and depression.

**Objective:**

This study aims to investigate the association between abuse and depression by investigating the mediating role of academic stress, social anxiety, and generalized anxiety to offer insights for depression prevention.

**Participants and setting:**

We investigated adolescents in urban and rural areas in Wansheng District of Chongqing city in China. This study included a total of 14,108 students from secondary and primary schools, among whom 7,086 were men (50.2%) and 7,022 were women (49.8%). The participants aged from 11 to 17 with an average age of 13.58 (*M* = 13.58, *SD* = 1.86).

**Methods:**

A questionnaire survey was conducted among the participants between October and December in 2020. The following questionnaires were adopted: DSM-5 Patient Health Questionnaire for Adolescents, DSM-5 Severity Measure for Generalized Anxiety Disorder, The International Society for Prevention of Child Abuse and Neglect Child Abuse Screening Tool, DSM-5 Severity Measure for Social Anxiety Disorder (Social Phobia), and Study Stress Scale.

**Results:**

The lifetime prevalence of abuse was high in Chinese adolescents. The direct effect value from abuse to depression was 0.05, the total mediating effect value was 0.14, and the overall effect value was 0.19. According to the mediating effect analysis, the direct and indirect effects from abuse to depression were significant, and the mediating effect accounted for 73.68% of the total effect, suggesting that 73.68% of the effects of abuse to depression are mediated by academic stress, social anxiety, and generalized anxiety.

**Conclusions:**

Academic stress, social anxiety and generalized anxiety play a chain multiple mediating role in the association between abuse and depression, which sheds light on the prevention of depression in the future.

## Introduction

Abuse event is common in people of all ages, ranging from children to the elderly (1, 2). Children are the major victims of abuse, which is intentional harm or a threat of harm to a child by someone acting in the role of caretaker, for even a short time. Maltreatment is commonly divided into four categories: physical abuse, sexual abuse, emotional abuse, and neglect (3). However, there is no specific legislation for child abuse in China. Although the incidence of child abuse decreased from 70.1% in 2010 (4) to 42.3–62.7% in 2019 (5) with the development of Chinese society, it is still much higher than that in western countries (e.g., 23.5% in New Zealand and 13.3% in Western Australia in 2015) (6). According to Article 260 of Criminal Law of the People's Republic of China, the crime of abuse can be categorized into physical abuse (beating, freezing and starving, confinement, forcing to work excessive hours, etc.) and psychological abuse (humiliating, swearing, an insult to dignity, etc.). Child abuse causes not only physical injury but also psychological trauma, and will even affect the mental health of the individual when he/she grows up (5, 7, 8). Studies have shown that parents are responsible for a majority of child abuse, which accounts for 81% of the total (9), indicating that family is the primary place where abuse takes place.

Depression, a complicated mental disorder, can lead to changes in emotional outcomes. Common emotions associated with depression include low mood, sadness, discomfort and distress. Somatic symptoms may also develop, such as sleeping problems and loss of appetite. The worst of it is that major depression can lead to suicide, or affect the social functions (10, 11). Depression usually starts in childhood (12, 13); thus, it is necessary to investigate its risk factors to implement early prevention. One previous study shows that childhood abuse is always associated with depression (14) and adolescents with abusive experiences are at a greater risk of depression (15).

Abuse can serve as an important predictor of depression: the greater number of abuse events one experiences, the more severe the depression (16). It is previously reported that compared with healthy controls, patients with major depression experience more severe psychological abuse and negligence (17). The childhood abuse can increase the risk of depression in adulthood (18), and frequent exposure to family abuse is an independent risk factor for adult depression (19). In addition, not only does abuse increase the risk of depression, but it also hinders the recovery from depression. One study shows that the recovery rate of women who were severely depressed but did not experience abuse is 3.7 times greater the rate of those with such experience in 12 months (20). Therefore, attention should be paid to psychological abuse from parents to reduce the risk of depression in adolescence (21). Those who experience abuse but have not experienced depressive symptoms yet should be the focus of attention for prevention to reduce the possibility of developing depression. Early intervention may be primary means for preventing worsening or recurrence of depressive symptoms. This study hypothesizes that a close association exists between abuse and depression, which may be mediated by three factors: academic stress, social anxiety and generalized anxiety.

Academic stress refers to the psychological burden and tension caused by stimuli related to learning activities (22). Appropriate academic stress is conducive to academic performance, while excessive stress fails to promote learning, and even leads to learning difficulties or decline in academic performance. In addition, it may impair mental health, resulting in school fear and truancy (23–25). An increase in academic stress among high school students (from 40% in 2006 to 42.62% in 2019) in China (26, 27) indicates that increasing attention should be paid to this issue. Academic stress stems from different sources, such as school, society, family and students themselves (22). Students with abusive experiences demonstrate greater academic stress. Multivariate logistic regression analysis showed that abusive experiences such as witnessing parents fighting (OR = 1.44), being bullied on campus (OR = 1.62), and being beaten and scolded by parents (OR = 2.32) are the main risk factors for academic stress (26). High self-perceived academic stress is an independent risk factor of depression (28). Meanwhile, negative stressful academic events (OR = 1.551, 95% CI: 1.207–1.993) are also a risk factor for depression (29). Therefore, increasing attention should be paid to the interactions among abuse, academic stress, and depression.

Abusive experiences are also associated with social avoidance (30), which is always considered as the core symptom of social anxiety, and a key standard for its diagnosis (31, 32). One previous study shows that adolescents with abusive experiences show more remarkable characteristics of social anxiety and avoidance (33), indicating that abuse is one risk factor for social anxiety. In addition, social anxiety will affect the interpersonal relationship, and in turn, depressive symptoms will develop or increase (34). Therefore, abusive experiences could affect the depression level of the individual *via* social anxiety.

Generalized anxiety, similar to social anxiety, is a type of anxiety disorder, but different from social anxiety, it is caused by worries about a variety of things (such as family, health, economic status, future, etc.). These worries are difficult to control, and are accompanied by non-specific psychological and physical symptoms (35). Abuse can lead to generalized anxiety and depression (36, 37), which are often comorbid and associated with each other (38). Generally speaking, most individuals with depression manifest symptoms of generalized anxiety. It is reported that 85% individuals with depression experience severe anxiety, and 90% anxiety individuals have depression at the same time in Australia (39), suggesting that generalized anxiety is a predictor of depression.

Because of the high rate of abuse in Chinese adolescents and the severe mental health problems associated with it (depression, anxiety, etc.), it is urgent to investigate the association between abuse and depression to shed light on the improvement of public strategy. This study makes the following hypotheses concerning the association between abuse, academic stress, social anxiety, generalized anxiety and depression: (1) Abuse can significantly predict depression among Chinese adolescents; (2) Academic stress, social anxiety, and generalized anxiety play a chain multiple mediating role in the association between abuse and depression. This study aims to investigate the role of academic stress, social anxiety and generalized anxiety in mediating the association between abuse and depression to provide rationale for preventing abuse in adolescents.

## Methods

### Study design

This study adopted a questionnaire on demographic information and a total of five scales: DSM-5 Patient Health Questionnaire for adolescents (PHQ-A) (40–43), DSM-5 Severity Measure for Generalized Anxiety Disorder (GAD-10) (40, 42, 44, 45), The International Society for Prevention of Child Abuse and Neglect Child Abuse Screening Tool (ICAST-C) (46–48), DSM-5 Severity Measure for Social Anxiety Disorder (Social Phobia) (SAD-10) (40, 42, 49), and Study Stress Questionnaire (SSQ) (22) to investigate the prevalence and frequency of depression, generalized anxiety disorder, abuse, social anxiety, and academic stress. Based on the findings of the scales, we conducted Spearman correlation analysis to analyze the correlation of the five variables. Then, after data standardization, we built and tested a chain multiple mediation model using structural equation modeling, in which abuse was used as an independent variable, depression a dependent variable, and academic stress, social anxiety and generalized anxiety as mediating variables.

### Research procedures

Paper questionnaires were distributed to the participants. Two postgraduates majoring in psychology trained the teacher of each class, and they together conducted the survey. In the classroom, one postgraduate student explained how to fill in the questionnaire to the participants, and then the two postgraduates answered the questions asked by the participants if they had any during completing the questionnaire. The class teacher provided assistance during the whole process. The participants were informed that they need to fill in the questionnaire honestly, and there is no right or wrong. They were ensured that their information would be kept confidential, and they could choose to quit at any time during the survey.

The current study was reviewed and approved by the Medical Ethics Committee of the Department of Medical Psychology, Army Medical University (No. CWS20J007). The approval from officials of sampled schools was obtained in a written form. Parents or legal guardians were contacted by the teacher to provide verbal consent on behalf of the children to participate in the study.

### Measures

#### Questionnaire on demographic information

Self-designed questionnaire was used to investigate the participants' demographic information (gender and age).

#### PHQ-A

PHQ-A based on Diagnostic and Statistical Manual of Mental Disorders V (DSM-5) was adopted to investigate the symptoms and degree of depression of the participants in the last 2 weeks, which is a widely used scale in clinical setting developed by American Psychiatric Association (APA) (42). Wang et al. translated the original scale into the Chinese version (40, 41), which has a high reliability. It contains nine items in total. The 4-point Likert scale was used, with the score ranging from 0 (none at all) to 3 (almost every day). The total score ranges from 0 to 27. According to previous studies, 0–4 means absence of depressive symptoms; 5–9 mild depressive symptoms; 10–14 moderate depressive symptoms; 15–19 moderate and severe depressive symptoms; and over 20 points severe depressive symptoms. The questionnaire has a good reliability in the current study with the Cronbach's α value of 0.89.

#### GAD-10

GAD-10 based on the DSM-5 was used to investigate the generalized anxiety level of the participants in the past 2 weeks, which is a widely used scale in clinical setting developed by APA (42) and was translated into the Chinese version by Meilihua Health Systems (40) and Zhang et al. (44) with a high reliability. It contains 10 items, such as sudden fear, fear and panic. The 5-point Likert scale was used, with the score ranging from 0 (never) to 4 (always). The total score ranges from 0 to 40. A higher score was associated with a higher level of anxiety and a larger number of anxiety symptoms. The same symptom classification criteria as in PHQ-A were adopted. The questionnaire has a good reliability in the current study with a Cronbach's α value of 0.90.

#### ICAST-C

ICAST-C (46–48) was used to investigate the abusive experiences of adolescents in the last year. It contains 36 items with five dimensions: violence exposure (7 items), emotional abuse (8 items), neglect (6 items), physical abuse (9 items), and sexual abuse (6 items). Because sexual abuse failed to pass the ethical review in the current study, it was excluded from the survey. The final survey contained the remaining 30 items. Participants were asked to give a score of 0–6 points for each item according to their experience in the past year (0: never happened, 1: happened but not in the past year, 2: 1–2 times, 3: 3–5 times, 4: 6–12 times, 5: 13–50 times, and 6: more than 50 times). The total score ranges from 0 to 180. A higher score was associated with a higher frequency and more types of abuse. In the current study, Cronbach' α value of the scale is 0.903 with 0.63 for violence exposure, 0.794 for emotional abuse, 0.761 for neglect and 0.789 for physical abuse.

#### SAD-10

SAD-10 based on the DSM-5 was used to assess the feelings and behaviors of the participants in social intercourse in the last 2 weeks, which was developed by APA (42) and is widely used in clinical setting. Wang et al. translated the original scale into the Chinese version (40), which has a high reliability. It contains 10 items, such as diversion of attention to avoid thinking of social intercourse. The 5-point Likert scale was adopted, with the score ranging from 0 (never) to 4 (always). The total score ranges from 0 to 40. A higher score was associated with a higher level of social anxiety or more social anxiety symptoms. The same symptom classification criteria as in PHQ-A were adopted. The questionnaire has a good reliability in the current study with a Cronbach's α value of 0.92.

#### SSQ

The SSQ for high school students (22), which is a Chinese scale, was used to investigate the study experience of the participants. It contains 21 items, assessing the stress from parents, oneself, teachers and social intercourse. Participants were asked to give a score of 1 (completely disagree) −5 points (completely agree) for each item. A higher score indicates higher academic stress. In this study, based on the score, we put the 50% students with a higher score (≥48) into the high-stress group, and the rest 50% (<48) into low-stress group. The questionnaire has a good reliability in the current study with a Cronbach's α value of 0.87.

### Statistical analysis

A total of 20,000 questionnaires were distributed to students from primary and secondary schools in Wansheng District of Chongqing city between October and December in 2020, and 18,133 were recovered. According to the scope of the questionnaire which is applicable to those aging between 11 and 17, the data of participants younger than 11 and older than 17 were excluded. The questionnaires which did not indicate gender or with a completion rate of <50% for any item were also excluded. At last, a total of 4,025 questionnaires were excluded, and the remaining 14,108 were included in the analysis. The average age of these participants was 13.58 (SD = 1.86).

The data were sorted and analyzed by Microsoft Excel, SPSS 25.0, R 4.0.0, and Mplus 8.3. First, Microsoft Excel was used for data preprocessing, including data cleaning and total score calculation. Then, SPSS 25.0 was used for common method bias testing and descriptive statistical analysis; R 4.0.0 for calculating the correlation; and Mplus 8.3 for constructing structural equation model and testing its mediating effect.

## Results

### Common method bias test

As the data were collected through self-reporting by participants, common method bias may exist. Therefore, Harman's single factor test was used to clarify whether bias existed for all variables. The results showed that there were 14 factors with non-rotating characteristic roots >1, explaining 56.45% of the total variance, and the first factor can explain 24.19% of the variation rate, which did not exceed the recommended critical value of 40%. Therefore, no common method bias existed in this current study.

### Descriptive statistics

Descriptive statistics of the questionnaires and scales are presented in Tables 1, 2. The descriptive analysis of the data of 14,108 participants showed that the participants aged from 11 to 17 (*M* = 13.58, *SD* = 1.86), including 7,086 men (50.2%) and 7,022 women (49.8%). In the current sample, the overall lifetime prevalence of abuse in adolescents is 88.63% (i.e., 68.51% within 1 year plus 20.12% in the past), indicating a high abuse prevalence in Chinese adolescents, as reported previously (4, 5). Among types of abuse, physical abuse is the most common, followed by exposure to violent scene and psychological abuse, and the prevalence of neglect is the lowest, suggesting that most adolescents have experienced different levels of physical abuse in the past, while less of them have been neglected (such as being starved, having no clothes, etc.). The prevalence of abuse in the last year indicates that the rate of exposure to violent scene is high, including witnessing family quarrels or fights. In addition, the prevalence of psychological abuse is as high as 48.01%, including insult and invective, verbal threat or ridicule. The prevalence of physical abuse and neglect is also high, being 36.30 and 31.31%, respectively (Table 3, Supplementary Table 1). As for the prevalence of depression, 6,035 participants (42.8%) reported different levels of depression, with 2,034 (14.4%) reporting moderate or severe depressive symptoms, while 8,073 (57.2%) reported absence of depressive symptoms.

**Table 1:** Characteristics of the participants (*n* = 14,108).

**Characteristics**	
Age, mean (SD), y	13.58 (1.86)
Male, *n* (%)	7,086 (50.2%)
Female, *n* (%)	7,022 (49.8%)

**Table 2:** Mean score and range for each variable.

	**M**	**SD**	**Range**
Abuse	13.88	15.52	0–168
Violence exposure	3.85	4.07	0–42
Emotional abuse	3.59	4.98	0–48
Neglect	3.16	4.80	0–36
Physical abuse	3.28	3.93	0–45
Generalized anxiety	5.91	6.70	0–40
Depression	5.09	5.31	0–27
Academic stress	48.57	14.41	21–105
Social anxiety	5.52	7.18	0–40

**Table 3:** The prevalence of different types of abuses in adolescents.

**Type of abuses**	**Never %**	**Occurred before but not in the last year%**	**1–50 times per year%**
Exposure to abuse scene	25.47 (26.84, 24.10)	20.66 (20.05, 21.28)	53.86 (53.10, 54.63)
Emotional abuse	29.72 (30.68, 28.75)	22.27 (22.64, 21.90)	48.01 (46.68, 49.34)
Neglect	53.21 (56.18, 50.21)	15.48 (15.95, 15.01)	31.31 (27.87, 34.78)
Physical abuse	23.26 (26.38, 20.11)	40.45 (38.88, 42.03)	36.30 (34.74, 37.87)
Total	11.37 (13.11, 9.61)	20.12 (19.45, 20.82)	68.51 (67.44, 69.57)

The value outside the parenthesis is the overall incidence of adolescents, while that in the parenthesis is the incidence of boys and girls, respectively.

### Correlation analysis

The correlations between the variables (abuse, academic stress, social anxiety, generalized anxiety, and depression) were analyzed using Spearman's rho. The analysis showed a significant positive correlation between those variables (Table 4).

**Table 4:** The results of correlation analysis of each variable.

	**Abuse**	**Academic stress**	**Social anxiety**	**Generalized anxiety**
Academic stress	0.41^***^			
Social anxiety	0.45^***^	0.38^***^		
Generalized anxiety	0.58^***^	0.40^***^	0.67^***^	
Depression	0.57^***^	0.43^***^	0.63^***^	0.76^***^

Spearman's rho was conducted to test the correlations between the variables.

*** P < 0.001.

### Abuse and depression in adolescents: Test of chain multiple mediation model

Collinearity diagnostics analysis was performed (50), which showed that all the tolerance values (0.45, 0.54, 0.62, and 0.77) were higher than 0.10, and variance inflation factors (VIFs) (2.22, 1.86, 1.61, and 1.29) were lower than 10. Multicollinearity did not exist.

**Figure 1:**
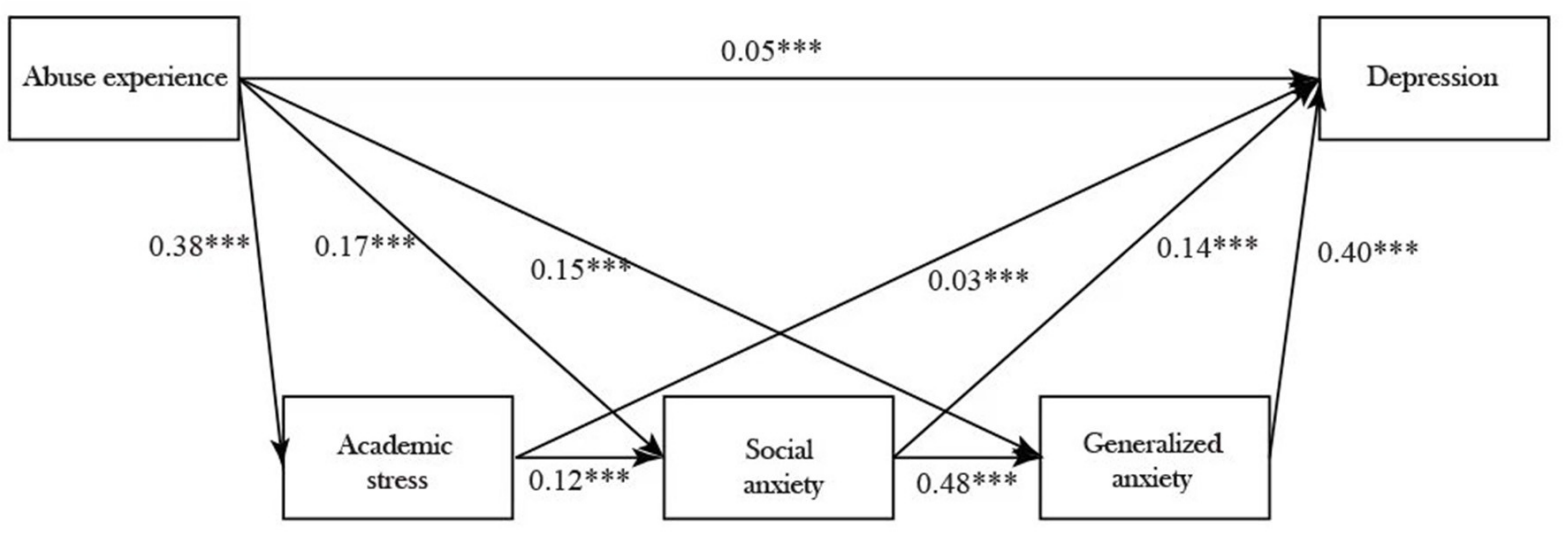
The multiple mediation model of abuse and depression. ***P < 0.001.

The correlation coefficient between variables was significant, which conforms to the requirement of mediation effect test. The chain multiple mediation model was tested using gender and age as control variables, abuse as an independent variable, depression as a dependent variable, and academic stress, social anxiety and generalized anxiety as mediating variables. All path coefficients in the model were significant (*P* < 0.001), and the results are shown in Figure 1.

The indexes for model fit were as follows: χ^2^/*df* = 60.89, CFI = 0.97, TLI = 0.96, SRMR = 0.03, RMSEA = 0.065. The current study adopted a large sample size, which may affect the value of χ^2^/*df* and RMSEA (51). Overall, these indexes indicate that the model fit is acceptable (52).

The percentile bootstrap method for bias correction was adopted to test the mediating effect at 95% confidence interval. A total of 5,000 samples were repeatedly and randomly selected from the original samples for model fitting. The results showed that the 95% confidence interval of each path did not contain zero, indicating that the effect of each path is significant (Table 5).

**Table 5:** The mediating effect of academic stress, social anxiety, and generalized anxiety in the association between abuse experience and depression.

**Path**	**Value of indirect effect**	**Boot standard error**	**95% confidence interval**	
			**Lower limit**	**Upper limit**
Path 1.Abuse experience—Academic stress—Depression	0.012	0.001	0.010	0.015
Path 2. Abuse experience—Social anxiety—Depression	0.023	0.002	0.020	0.026
Path 3. Abuse experience—Generalized anxiety—Depression	0.061	0.002	0.056	0.066
Path 4. Abuse experience—Academic stress—Social anxiety -Depression	0.006	0.001	0.005	0.007
Path 5. Abuse experience—Social anxiety—Generalized anxiety—Depression	0.032	0.001	0.029	0.034
Path 6. Abuse experience—Academic stress—Social anxiety—Generalized anxiety—Depression	0.008	0.001	0.008	0.010
The total indirect effect value: Abuse experience—Depression	0.142	0.003	0.136	0.149

The direct effect value from abuse to depression was 0.05, the total mediating effect value was 0.14 (i.e., the sum of the effect values of the six intervening paths), and the total effect value was 0.19 (i.e., the direct effect value plus the total mediating effect value) (53). The mediating effect analysis showed that the direct and indirect effects from abuse to depression were significant, and the mediating effect accounted for 73.68% of the total effects, suggesting that 73.68% of the effects of abuse to depression are mediated by academic stress, social anxiety and generalized anxiety. The effect values of the six paths accounted for 6.31, 12.10, 32.11, 3.16, 16.84, and 4.21% of the total effect values, respectively.

## Discussion

### The prevalence of abuse in adolescents

As shown in Table 3, many adolescents are experiencing different degrees of abuse currently. On the one hand, this may be attributed to ways of education in China, such as physical punishment adopted by parents (54, 55), which may increase the risk of physical abuse. On the other hand, this may be explained by a lack of understanding of abuse. Parents in different cultures have different definitions of neglect or psychological abuse (56); many do not consider psychological abuse or neglect as a form of abuse. Therefore, it is important to formulate corresponding policies to lower the high prevalence of abuse in Chinese adolescents.

### Correlation between the variables

The correlation analysis shows a significant positive correlation between abuse, academic stress, social anxiety, generalized anxiety and depression. Previous studies have reported the association between two or three of these variables. For example, Lin et al. suggested that violent behaviors from the parents may lead to psychological trauma of the children, resulting in increased academic stress (26); Adams et al. found a significant association of childhood physical abuse with depression and anxiety (57); and Shapero et al. reported a possible association between severity of emotional abuse and depressive symptoms in adults (58). However, to our knowledge, this is the first study to investigate the relation of these five variables and the mediating role of academic stress, social anxiety, generalized anxiety in the association between abuse and anxiety, which enriches the current understanding of the mechanism underlying how abuse might contribute to the development of depression.

### The mediating effect of academic stress, social anxiety, and generalized anxiety in the association between abuse and depression in adolescents

The results of this study show that abusive experiences in adolescents are significantly positively correlated with depression, and the level of abuse can significantly predict the severity of depression. Academic stress, social anxiety and generalized anxiety play a role of chain multiple mediation in the relation between abuse and depression, with six paths of mediating effect (Table 5).

The effect value of “path 6” accounts for 4.21% of the total effect. Abuse occurs because of academic stress under many circumstances, such as homework tutoring or low examination scores (59–61). Research shows that abuse significantly differs among different levels of academic achievements, and is positively correlated with academic stress (62, 63). The adolescents who are abused because of their academic achievement will seek to avoid being abused again next time because of the same reason, resulting in the development of academic stress. Stress theory holds that stress or stress events will lead to changes in biological system, affecting brain and behavior, and neuroendocrine and immune systems, reducing stress coping ability of the individual. Exposure to acute and chronic stress can cause anxiety, a common neurobehavioral factor of various stress sources (64). Long-term academic stress experienced by the adolescents may upset their immune balance, resulting in increased anxiety (26).

High academic stress will also lead to avoidance of relevant scenes, including schools, teachers, or classmates, and this social avoidance is a core symptom of social anxiety. Social avoidance also occurs because abusive experiences destroy secure attachment. According to the attachment theory, whether a secure attachment can be formed between children and their guardians will affect the development of the safety internal working model, as well as the future development of relationship with peers or lovers (65). In addition, the theory of the long-term effect of child abuse shows that abused children do not receive sufficient guardian protection, nor do they constitute the internal representation of an effective protector. Therefore, they have difficulties in resisting interpersonal attacks and internal self-criticism, resulting in interpersonal problems (66).

In this study, the lifetime prevalence of abuse in adolescents is as high as 88.63%. When the adolescents face the abuse by their guardians, they usually seek to meet the requirements of the latter (such as better academic performance) in order to avoid further abuse. Therefore, individuals have to make greater effort, leading to increased stress. As a result, they try to avoid stress-related environment (such as school, classmates); the social avoidance can eventually lead to social anxiety. Meanwhile, the increased level of social avoidance indicates that adolescents are unable to obtain effective social support when facing stress, especially support from their parents (67). As social support can confer resilience to stress (68), a lack of it makes people unable to deal with the stress and prone to develop excessive and uncontrollable worries, which are the characteristics of generalized anxiety disorder (69). As the anxiety builds up, depression will eventually develop, thus forming the path of “abuse—academic stress—social anxiety—generalized anxiety—depression.”

### Research contributions and limitations

This study discusses the association between abuse and depression in adolescents, and confirms the multiple mediating effects of academic stress, social anxiety, and generalized anxiety on the association between the two, which provides not only theoretical mechanism underlying adolescent depression, but also insights for the prevention of adolescent depression. The prevalence of abuse in children and adolescents is high in Chinese society; however, there is a lack of studies on adolescent abuse previously. Therefore, research on abuse in adolescents is of practical significance for protection of adolescents. Our results could alert the parents to the damages that can be brought by different types of abuse (violence exposure, emotional abuse, neglect, or physical abuse), thus reminding them about their improper ways of parenting. In addition, the results may help the schools realize their important role in mediating the relation between abuse and depression by adopting necessary measures, such as providing counseling service or initiating mental health activities to alleviate anxiety of the abused students. Furthermore, the research results can provide support for the government to formulate new policies and guidance for parents to improve their educational methods. By attracting the attention of the parents, schools, and governments to the mental problems associated with abuse, our study may hopefully promote the academic achievement as well as physical and mental development of the adolescents.

This study has several limitations. Firstly, this study does not consider personality, which is an important factor for perceived academic stress. In the future study about the mental health of adolescents, this factor needs to be included (70). Secondly, as this is a cross-sectional survey, causal inference cannot be made based on the results. In particular, there may be a two-way or simultaneous interaction between generalized anxiety and depression. Therefore, longitudinal association between the two should be further explored in the future. Thirdly, although the study adopts large sample survey, it does not include samples from multiple regions. In China, a nation with many cultural branches, generalization of the results may be limited. For example, as there may be differences in family rearing patterns between developed areas and rural underdeveloped areas, abuse may be classified into different types. In the future, the results should be confirmed by national survey with a larger sample. Finally, due to the scope of the survey tools, this study excludes the data of participants who do not meet the age requirement. Although this meets the theoretical requirements, a lot of useful information may get lost. In this regard, we can explore the validity of data around age critical point in the future to extend the scope of survey tools.

## Conclusion

In summary, the lifetime prevalence of abuse is very high in Chinese adolescents and academic stress, social anxiety and generalized anxiety play a role of chain multiple mediation between abuse and depression. This study not only provides theoretical mechanism underlying the association between abuse and depression in adolescents, but also insights for the prevention of associated mental illness.

## Data availability statement

The original contributions presented in the study are included in the article/Supplementary material, further inquiries can be directed to the corresponding authors.

## Ethics statement

The studies involving human participants were reviewed and approved by Medical Ethics Committee of the Department of Medical Psychology, Army Medical University. Written informed consent from the participants' legal guardian/next of kin was not required to participate in this study in accordance with the national legislation and the institutional requirements.

## Author contributions

XZ: conceptualization, validation, and writing—original draft. KL: methodology, software, and data analysis. YZ: data curation and visualization. GY: supervision. XL: supervision and writing—review and editing. All authors contributed to the article and approved the submitted version.

## Conflict of interest

The authors declare that the research was conducted in the absence of any commercial or financial relationships that could be construed as a potential conflict of interest.

## Publisher's note

All claims expressed in this article are solely those of the authors and do not necessarily represent those of their affiliated organizations, or those of the publisher, the editors and the reviewers. Any product that may be evaluated in this article, or claim that may be made by its manufacturer, is not guaranteed or endorsed by the publisher.
