# Supplementary material for: A chain multiple mediation model reveals the association between abuse and depression in Chinese adolescents
Source: Front Public Health. 2022 Nov 17;10:1023749. doi: 10.3389/fpubh.2022.1023749 (PMC9714438; doi:10.3389/fpubh.2022.1023749)
Supplement: Supplementary file 1 [file Table_1.DOCX]

Supplementary Table 1 The incidence of each abuse event in adolescents

| Abuse events | Never% | Occurred before but not in the last year% | 1-2 times per year% | 3-5 times per year% | 6-12 times per year% | 13-50 times per year% | Over 50 times per year% |
| --- | --- | --- | --- | --- | --- | --- | --- |
| Frightened by adults’ using drugs | 69.8 (71.2, 68.3 | 16.7 (16.2, 17.2) | 7.3 (6.9, 7.6) | 3.6 (3.3, 3.8) | 1.4 (1.3, 1.5) | 0.8 (0.6, 1.0) | 0.4 (0.4, 0.5) |
| Adults shouting in frightening way | 87.0 (87.9, 86.2) | 8.2 (7.6, 8.9) | 2.6 (2.5, 2.6) | 1.3 (1.1, 1.5) | 0.5 (0.6, 0.4) | 0.3 (0.2, 0.3) | 0.1 (0.2, 0.1) |
| Witnessing adults in home hitting, kicking, slapping | 95.5 (96.4, 94.7) | 3.0 (2.4, 3.6) | 0.8 (0.6, 0.9) | 0.3 (0.2, 0.4) | 0.1 (0.1, 0.2) | 0.1 (0.1, 0.1) | 0.1 (0.1, 0.1 |
| Witnessing adults in home using weapons | 93.9 (94.5, 93.2) | 4.3 (3.8, 4.9) | 1.1 (1.0, 1.1) | 0.4 (0.2, 0.5) | 0.2 (0.2, 0.1) | 0.1 (0.2, 0.1) | 0.1 (0.1, 0.1) |
| Someone close got killed near home | 86.5 (87.9, 85.1) | 10.6 (9.1, 12.1) | 2.1 (2.1, 2.2) | 0.5 (0.5, 0.4) | 0.1 (0.2, 0.1) | 0.1 (0.1, 0.1) | 0.0 (0.1, 0.0) |
| Having seen people being shot or rioting | 52.2 (53.1, 51.3) | 16.8 (16.7, 16.9) | 11.3 (10.8, 11.8) | 6.9 (6.6, 7.2) | 4.5 (4.5, 4.6) | 4.4 (4.3, 4.5) | 3.8 (3.9, 3.7) |
| Something stolen from home | 46.8 (47.5, 46.1) | 16.3 (16.4, 16.2) | 11.0 (10.5, 11.4) | 8.7 (8.1, 9.3) | 5.2 (5.5, 4.9) | 5.6 (4.9, 6.3) | 6.5 (7.2, 5.8) |
| Screaming | 51.3 (53.4, 49.2) | 15.8 (15.3, 16.3) | 11.8 (11.3, 12.3) | 9.0 (8.2, 9.9) | 4.4 (4.5, 4.4) | 4.3 (4.1, 4.6) | 3.3 (3.4, 3.3) |
| Insulted | 68.8 (70.8, 66.8) | 11.7 (11.2, 12.2) | 8.2 (7.5, 8.9) | 5.6 (5.2, 5.9) | 2.5 (2.4, 2.6) | 2.1 (1.9, 2.3) | 1.1 (1.0, 1.3) |
| Feeling embarrassed | 80.9 (82.7, 79.1) | 8.9 (8.1, 9.6) | 4.4 (4.1, 4.8) | 3.0 (2.5, 3.5) | 1.3 (1.2, 1.4) | 1.0 (0.9, 1.1) | 0.5 (0.4, 0.5) |
| Wished you were dead | 81.6 (82.3, 80.9) | 11.6 (11.1, 12.2) | 3. 9 (3.9, 3.9) | 1.6 (1.7, 1.6) | 0.6 (0.4, 0.8) | 0.4 (0.4, 0.5) | 0.2 (0.3, 0.2) |
| Threatened to abandon | 78.0 (80.4, 75.5) | 11.5 (10.8, 12.3) | 4.9 (4.5, 5.3) | 2.7 (2.1, 3.3) | 1.3 (0.9, 1.7) | 0.9 (0.7, 1.2) | 0.7 (0.6, 0.8) |
| Locked out of home | 89.6 (90.5, 88.7) | 4.7 (4.4, 5.1) | 2.7 (2.7, 2.7) | 1.4 (1.2, 1.6) | 0.6 (0.4, 0.8) | 0.6 (0.5, 0.7) | 0.4 (0.3, 0.5) |
| Bullied by another child at home | 95.3 (95.9, 94.7) | 1.7 (1.6, 1.8) | 1.5 (1.3, 1.7) | 0.6 (0.5, 0.8) | 0.2 (0.2, 0.2) | 0.3 (0.2, 0.4) | 0.2 (0.2, 0.3) |
| Being hungry or thirsty | 92.6 (94.1, 91.1) | 3.2 (2.9, 3.6) | 2.2 (1.7, 2.7) | 1.1 (0.7, 1.5) | 0.5 (0.3, 0.7) | 0.2 (0.2, 0.2) | 0.2 (0.2, 0.2) |
| Inadequate clothing | 85.7 (88.2, 83.1) | 5.3 (4.9, 5.7) | 4.1 (3.1, 5.0) | 2.3 (1.8, 2.8) | 1.2 (1.0, 1.4) | 0.8 (0.5, 1.2) | 0.6 (0.5, 0.8) |
| Unmet medical need | 76.9 (82.0, 71.8) | 7.5 (6.7, 8.3) | 6.2 (4.7, 7.8) | 4.0 (2.8, 5.2) | 1.9 (1.3, 2.4) | 1.7 (1.2, 2.2) | 1.8 (1.4, 2.3) |
| Feeling not cared for | 71.4 (76.3, 66.5) | 9.3 (8.4, 10.2) | 7.6 (5.4, 9.7) | 4.8 (3.9, 5.6) | 2.6 (2.3, 2.9) | 2.3 (1.9, 2.7) | 2.1 (1.9, 2.4) |
| Feeling unimportant | 87.6 (88.6, 86.5) | 4.8 (4.5, 5.0) | 3.2 (2.8, 3.5) | 1.9 (1.6， 2.2) | 1.0 (0.8， 1.2) | 0.7 (0.8， 0.6) | 0.9 (0.9， 0.9) |
| Inadequate support/help | 76.5 (75.4，77.7) | 12.8 (13.6, 12.0) | 4.8 (5.2, 4.4) | 3.3 (3.1, 3.6) | 1.2 (1.2, 1.2) | 0.7 (0.6, 0.8) | 0.6 (0.8, 0.4) |
| Threatened to hurt or kill you | 57.4 (57.0, 57.9) | 24.6 (25.2, 24.0) | 8.1 (8.4, 7.9) | 5.7 (5.3, 6.2) | 2.1 (2.2, 2.1) | 1.3 (1.3, 1.4) | 0.6 (0.7, 0.6) |
| Pushed, grabbed, kicked | 54.0 (52.5, 55.6) | 29.3 (29.8, 28.7) | 8.1 (8.5, 7.6) | 5.1 (5.5, 4.7) | 2.0 (2.0, 1.9) | 1.0 (1.1, 0.8) | 0.6 (0.6, 0.6) |
| Hit, beat, spanked with hand | 83.1 (82.2, 84.0) | 10.3 (11.0, 9.6) | 3.4 (3.6, 3.1) | 1.8 (1.7, 2.0) | 0.7 (0.7, 0.7) | 0.5 (0.5, 0.4) | 0.2 (0.2, 0.2) |
| Hit, beat, spanked with object | 97.6 (97.6, 97.7) | 1.5 (1.3, 1.6) | 0.5 (0.6, 0.4) | 0.2 (0.2, 0.2) | 0.1 (0.1, 0.1) | 0.0 (0.0, 0.0) | 0.0 (0.1, 0.0) |
| Trying to choke, smother, or drown | 95.9 (95.8, 96.1) | 2.7 (2.8, 2.6) | 0.8 (0.9, 0.7) | 0.3 (0.3, 0.3) | 0.1 (0.1, 0.2) | 0.1 (0.2, 0.1) | 0.1 (0.1, 0.1) |
| Burned or scalded | 77.8 (77.4, 78.2) | 12.8 (13.0, 12.5) | 4.3 (4.3, 4.3) | 2.9 (2.8, 3.0) | 1.2 (1.2, 1.1) | 0.7 (0.7, 0.6) | 0.4 (0.5, 0.3) |
| Locked in small place | 84.6 (83.7, 85.5) | 8.2 (8.4, 8.0) | 3.5 (3.5, 3.5) | 2.0 (2.4, 1.6) | 0.8 (1.0, 0.6) | 0.6 (0.7, 0.4) | 0.4 (0.4, 0.4) |
| Pulled hair, pinched, twisted ear | 92.8 (92.7, 92.8) | 4.1 (4.1, 4.1) | 1.6 (1.6, 1.5) | 0.9 (0.7, 1.1) | 0.3 (0.5, 0.2) | 0.2 (0.2, 0.2) | 0.2 (0.2, 0.1) |
| Holding heavy load as punishment | 69.2 (74.5, 63.7) | 22.9 (19.5, 26.4) | 4.3 (3.3, 5.2) | 2.0 (1.5, 2.6) | 1.5 (1.0, 2.1) | 0.0 (0.1, 0.0) | 0.0 (0.0, 0.0) |
| Threatened with a knife or stick | 43.2 (50.7, 35.6) | 39.2 (36.1, 42.2) | 10.7 (8.0, 13.3) | 3.7 (2.9, 4.6) | 3.2 (2.2, 4.3) | 0.0 (0.0, 0.0) | 0.0 (0.0, 0.0) |

The value outside the parenthesis is the overall incidence of adolescents, while that in the parenthesis is the incidence of boys and girls, respectively.
